# Systemic Mechanisms of Brain Rejuvenation

**DOI:** 10.1093/geroni/igab046.754

**Published:** 2021-12-17

**Authors:** Saul Villeda

**Affiliations:** University of California San Francisco, San Francisco, California, United States

## Abstract

Aging drives cellular and cognitive impairments in the adult brain. It is imperative to gain mechanistic insight into what drives aging phenotypes in the brain in order to maintain, and even restore, functional integrity in the elderly. We, and others, have shown that systemic manipulations - such as heterochronic parabiosis (in which a young and old circulatory system are joined) and administration of young blood or exercise induced blood factors - can reverse age-related impairments in regenerative, synaptic and inflammatory processes, as well as rescue cognitive faculties in the aged brain. These studies have revealed an age-dependent bi-directionality in the influence of the systemic environment indicating pro-youthful factors in young blood elicit rejuvenation while pro-aging factors in old blood drive aging. It has been proposed that introducing pro-youthful factors or mitigating the effect of pro-aging factors may provide effective strategies to rejuvenate aging phenotypes in the brain. Despite this potential, much is unknown as to the systemic and molecular mechanisms regulating pro-youthful and pro-aging effects of blood-borne factors. I will discuss work from my research group that begins to provide mechanistic insight into the systemic and molecular drivers promoting rejuvenation in the aging brain.

